# A novel *SCN9A* splicing mutation in a compound heterozygous girl with congenital insensitivity to pain, hyposmia and hypogeusia

**DOI:** 10.1111/jns.12280

**Published:** 2018-07-23

**Authors:** Margherita Marchi, Vincenzo Provitera, Maria Nolano, Marcello Romano, Simona Maccora, Ilaria D'Amato, Erika Salvi, Monique Gerrits, Lucio Santoro, Giuseppe Lauria

**Affiliations:** ^1^ Neuroalgology Unit IRCCS Foundation “Carlo Besta” Neurological Institute Milan Italy; ^2^ Neurology Department Istituti Clinici Scientifici Maugeri, IRCCS Telese Terme Benevento Italy; ^3^ Department of Neurosciences, Reproductive Sciences and Odontostomatology University Federico II Naples Italy; ^4^ Department of Neurology AOOR Villa Sofia Cervello Palermo Italy; ^5^ IRCCS “Bonino‐Pulejo” Messina Italy; ^6^ Department of Health Sciences University of Milan Milan Italy; ^7^ Clinical Genetics and Department of Neurology Maastricht University Medical Center Maastricht The Netherlands; ^8^ Department of Biomedical and Clinical Sciences “Luigi Sacco” University of Milan Milan Italy

**Keywords:** congenital insensitivity to pain, skin biopsy, small fiber neuropathy, sodium channel

## Abstract

Congenital insensitivity to pain (CIP) is a rare autosomal recessive disorder presenting with a spectrum of clinical features caused by mutations in different genes. A 10‐year‐old girl with CIP, hyposmia and hypogeusia, and her unaffected twin and parents underwent next generation sequencing of *SCN9A* exons and flanking splice sites. Transcript analysis from whole blood successfully assayed the effect of the mutation on the mRNA splicing by polymerase chain reaction amplification on cDNA and Sanger sequencing. We identified the novel splicing variant c.1108‐2A>G compound with the p.Arg896Gln (c.2687G>A) missense mutation previously described in a homozygous patient. The new intronic variant was predicted to induce exon 10 skipping. Conversely, SCN9A mRNA assay demonstrated its partial deletion with a loss of 46 nucleotides causing a premature stop codon in position p.Gln369 (NP_002968). Genetic analysis showed that the two variants were biallelic, being the mother and brother heterozygous carriers of the missense mutation, and the father heterozygous for the splicing mutation. Skin biopsy showed lack of Meissner's corpuscles, loss of epidermal nociceptors and normal autonomic organ innervation. We report a novel splicing mutation and provide clues on its pathogenic effect, broadening the spectrum of genotypes and phenotypes associated to CIP.

## 1 INTRODUCTION

Congenital insensitivity to pain (CIP) is a rare autosomal recessive syndrome encompassing a spectrum of clinical features that include hyposmia or anosmia, mental retardation, fever episodes, anhydrosis and other autonomic dysfunctions. Central to the disorder is the inability of affected subjects to transmit potentially noxious stimuli throughout the nociceptive pathway. The lack of pain perception and, therefore, of its awareness, leads to the accumulation of injuries, bruises, bone fractures, and other tissue injuries such as tongue or finger wounds due to self‐biting and multiple burns, which contribute in reducing life expectancy of affected children. Mutations in different genes essential for pain sensing have been described are patients harboring party different phenotypes.^1^

The first description of a person who apparently did not feel any pain except headache was published in 1932.^2^ More structured clinical descriptions, including anhydrosis and mental retardation, date back to the 1950s' of the 20th century,^3^ about 50 years before the discovery of the first causative mutations in *NTRK1*, the gene encoding a tyrosine receptor for nerve growth factor.^4^ The syndrome was classified as hereditary sensory and autonomic neuropathy (HSAN) type IV or CIP and anhydrosis (OMIM: 256800), and the role of *NTRK1* was later elucidated.^5^ Mutations in *NGFB* have been associated with childhood or adult onset of insensitivity to pain and classified as HSAN type V.^1^

A phenotype most commonly limited to insensitivity to pain and anhydrosis, with invariably normal intelligence, has been associated to loss‐of‐function mutations, principally nonsense, of *SCN9A* encoding the 1.7 sodium channel α subunit (OMIM: 603415).^6^ Evidence of the genetic and phenotypic heterogeneity of the syndrome, also named channelopathy‐associated insensitivity to pain (OMIM: 243000), came thereafter with the identification of further *SCN9A* mutations.^7^, ^8^, ^9^, ^10^, ^11^, ^12^, ^13^, ^14^, ^15^, ^16^ In these patients, large neuron‐dependent sensory modalities, such as touch and proprioception, are unaffected or mildly affected as shown by sural nerve biopsy and nerve conduction studies.^17^, ^18^ Hyposmia or anosmia, hypogeusia, bone dysplasia, and variable autonomic dysfunctions can enrich the clinical picture.^18^, ^19^ More recently, a gain‐of‐function mutation in *SCN11A* encoding the 1.9 sodium channel α subunit has been found to segregate with the phenotype of insensitivity to pain, anosmia, hyperhidrosis, gastrointestinal dysfunction, mild muscular weakness and, in some patients, intense pruritus.^20^

A small number of inactivating mutations in *SCN9A* have been published and, among these, truncating variants due to nonsense mutations or small ins/del represents the majority. We describe, providing evidence of the pathogenic effect and pathological details widening the phenotype, a novel splicing mutation biallelic to a known missense mutation in *SCN9A* found in a young patient with pain insensitivity and hyposmia.

## 2 METHODS

### 2.1 Clinical picture

**Figure 1:**
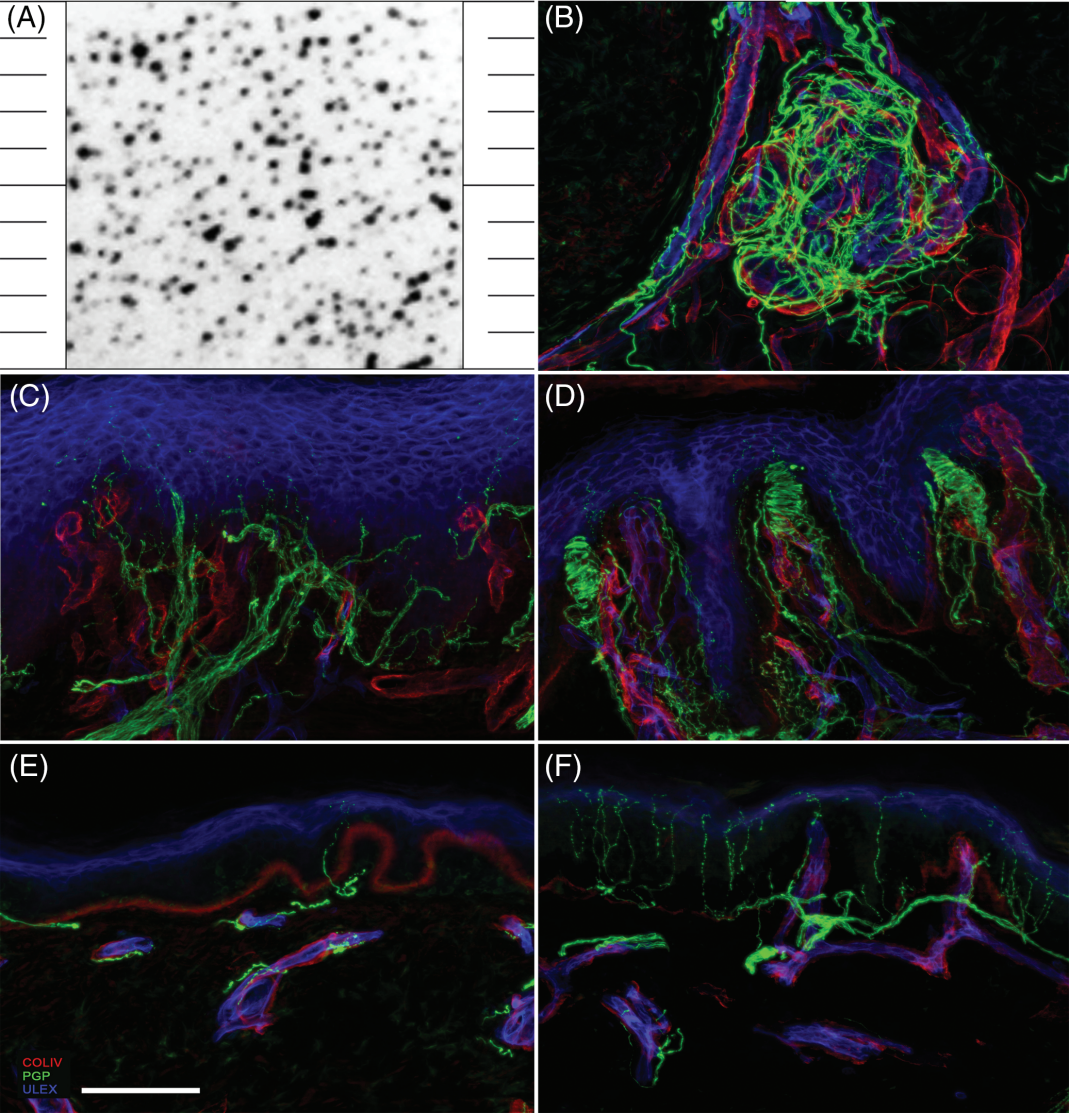
A, Shows the normally dense and homogeneous pattern of sweat drop imprints obtained in the patients after pilocarpine stimulation by dynamic sweat test. Consistently, B shows the normal pattern of sweat gland innervation by post‐ganglionic sudomotor nerves. Confocal micrographs of glabrous (C, D) and hairy (E, F) skin biopsy sections from the patient (C, E) and an age‐matched healthy subject (D, F). In C, note the complete lack of Meissner corpuscles and in E the severe reduction of intraepidermal nerve fiber density, compared to D and F, respectively. In green, nerve fibers marked with the pan neuronal marker protein‐gene‐product 9.5; in red, blood vessels and basement membranes marked with collagen IV; in blue, endothelia and keratinocytes marked with ULEX europaeus. Scale bar equals 100 μm

The proband was a young girl born pre‐term at the 36th week of pregnancy, twin of a heterozygous brother from non‐consanguineous parents. She did not show dysmorphic features and had a regular motor and intellectual development. At the age of 2 years, she started biting her tongue, hands and feet, and could near her fingers to the flame of a candle without discomfort. The neurological examination was normal except for the evidence that she did not retract when stimulated with a pricking pin, what was even clearer when punctured for blood tests. Due to her young age, quantitative sensory testing (QST), a psychophysical examination requiring the full compliance of the subject, could not be performed. Nevertheless, she perceived normally tactile and vibratory stimuli at the clinical evaluation. Sensory and motor nerve conduction study showed normal amplitude and velocity of upper and lower limb nerves. The dynamic sweat test (DST) was normal indicating an efficient sweat production, and dense and homogeneous pattern of activated sweat glands (Figure 1A). Skin biopsy showed that autonomic structures maintained a normal innervation (Figure 1B) whereas there was a complete lack of Meissner's corpuscles (Figure 1C,D) and a severe reduction of epidermal nerve fiber density (1.6/mm) (Figure 1E,F).

Over the years, the patient attended brilliantly the school and lived a fairly normal life also thanks to the familiar support. Even though she learnt how to avoid accidental traumas and self‐inflicted injuries, she experienced the painless fracture of the elbow bone and several episodes of purulent otitis with tympanic membrane perforation. She was re‐evaluated at the age of 8, when hyposmia and hypogeusia become apparent. DST showed the regular recruitment of sweat glands and normal production of sweat after pilocarpine by iontophoresis confirming that sudomotor function was not affected. QST showed a normal perception of tactile stimuli tested using Semmes Weinstein calibrated monofilaments and normal thresholds for cold and warm innocuous stimuli tested by a computer‐assisted system (Thermal sensory analyzer, TSA 2001 ‐ Medoc, Medoc Ltd., Ramat Yishai, Israel) equipped with a 3 × 3 cm Peltier device using the method of limits. She was able to discriminate between mechanical stimuli delivered with blunt or sharp probes, whereas pinprick test did not elicit pain. Follow‐up skin biopsy confirmed the severe reduction of epidermal nerve fibers (0.8/mm) and the lack of Meissner's corpuscles, with retained autonomic dermal annexes innervation. Genetic tests ruled out mutations in the exon 17 of NTRK1 gene (cr. 1q23‐q31) and in the coding exon of NGFB gene (cr.1p13.1).

### 2.2 SCN9A mutation screening and mRNA assay

Patient, her twin and parents' genomic DNA were extracted from peripheral blood sampling with Puregene Blood Kit (QIAGEN S.r.l., Milan, Italy), according to manufacturer instructions. Genetic analysis was performed on proband sample by a molecular inversion probe‐NGS (MIP‐NGS) approach, sequenced on HiSeq 2500 Illumina platform. Single molecule MIPs were designed to capture the coding and exon‐flanking intron sequences of nine sodium channel genes expressed in the nociceptive pathway. Relying on the association between loss‐of‐function mutations in the SCN9A gene (HGNC:10597) and the phenotype presented, this gene was first investigated. Both NGS results confirmation and the inheritance pattern were assessed by Sangers sequencing, using a primer pair covering exon 10 and its flanking splice sites (CATGCCTGTCAAATTGAAATA and CCGTTTGCATTTCTACCTCT). Amplicons were purified with IllustraExoProStar 1‐Step (GE Healthcare, Milan, Italy) and bi‐directionally sequenced using Big Dye Terminator v1.1Cycle Sequencing Kit on a 3130xl Genetic Analyzer System (Applied Biosystems, Thermo‐Fisher, Waltham, MA, USA). Splicing consequences were tested on the SCN9Atranscript (NM_002977.3). Total RNA of the proband was extracted from peripheral blood collected in a PaxGene Tube, using the PaxGene RNA extraction kit (all by Qiagen), according to manufacturer instructions. Retrotranscription was obtained by using SuperScript VILO cDNA Synthesis Kit (Invitrogen, Waltham, MA, USA), cDNA was amplified in two different polymerase chain reaction, using a forward primer hybridizing exon 9 and two different reverse primers, spanning either till exon 10 or to exon 11 (9F: ACTTTCAGCTGGGCCTTCTT; 10R: CAACCACAGCCAGGATCAAG; 11R: TGAGAGGCCCATAATTCTGCT). cDNA amplicons were separated on a low‐melting agarose gel, different sized fragments were cropped out, purified with PureLink Quick Gel Extraction Kit (Invitrogen) and then sequenced by Sanger technique as mentioned above. Splicing mutation prediction was assessed by Alamut software, provided by Interactive‐biosoftware (Rouen, France) under license.

## 3 RESULTS

**Figure 2:**
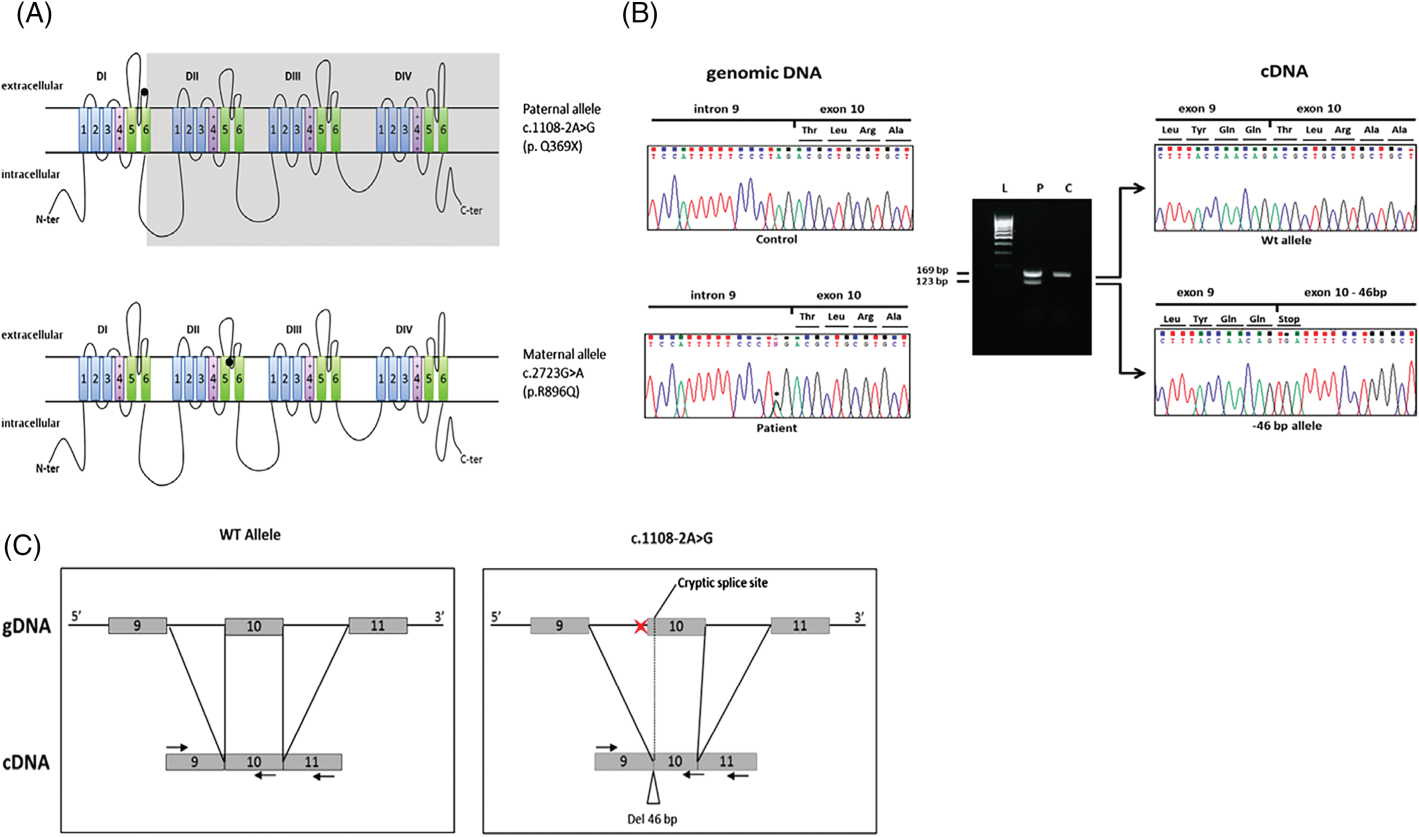
A, Localization of the two null mutations in Nav1.7 structure. The splicing mutation c1108‐2A>G on the paternal allele induces the premature truncation from the sixth segment of the first domain (S6‐DI). The missense mutation on the maternal allele is localized in the pore‐delimiting region. B, Proband SCN9A genomic sequencing, on the left side, revealed a nucleotide substitution in position c.1108‐2. The run on agarose gel show different bands from the SCN9A transcript amplification: in the patient lane (P) there is an unexpected fragment compared to the healthy control (C). Sanger sequencing of the two bands, on the right side, revealed the deletion of 46 nucleotides as a consequence of the canonical splicing site abolition and the new splicing‐acceptor site recognition. C, The splicing mutation on the genomic DNA and cDNA led to the deletion of 46 nucleotides on the mature transcript. Black arrows indicate the primer pair used for the cDNA amplification and sequencing

After the application of standardized pipeline for the analysis of MIP‐NGS data output, two single nucleotide variants in SCN9A gene (NM_002977.3) were identified (Figure 2A): a missense mutation in exon 16 leading to a substitution of the arginine residue in position 896 with a glutamine (p.Arg896Gln), and a nucleotide substitution affecting the exon 9 splice acceptor site (c.1108‐2A>G). Inheritance testing demonstrated the biallelic condition. SCN9A gene sequencing on the unaffected family members showed the heterozygous missense mutation in the mother and the brother, whereas the father harbored the heterozygous splicing mutation. The proband transcript analysis was carried out by the amplification of the exonic region interested by the splicing mutation and revealed the presence of an unexpected amplicon band, 46 bp shorter than the control, and the WT allele bands (Figure 2B). Sanger sequencing of the shorter fragment showed a 46 bp deletion indicating the delocalization of intron 9 splice acceptor site 46 nucleotides downstream within the exon 10 (Figure 2C).

## 4 DISCUSSION

We reported a new *SCN9A* splicing mutation in a compound heterozygous young patient presenting with CIP, hyposmia, and hypogeusia. The clinical features resembled those observed in the majority of patients previously reported as affected by loss‐of‐function *SCN9A* mutations. Indeed, our patient showed normal intelligence and autonomic functions, and had no dysmorphisms.

The missense mutation p.Arg896Gln localizes in the transmembrane region that delimits the channel pore in the second domain (S5‐S6/DII). It was previously identified in a homozygous CIP patient from consanguineous Bedouin parents and reported to have to a loss‐of‐function effect on the protein.^8^ This study highlighted the importance of the aminoacid sequence delimiting the pore region, which is highly conserved between orthologous and paralogous genes, not only for the electrophysiological properties of the channel, but also for its correct folding and surface localization. Recently, this variant has been reported in dbSNP (rs1024152367) with a minor allele frequency of 8 x 10^−6^ (www.ncbi.nlm.nih.gov/projects/SNP).

The novel intronic variant c.1108‐2A>G found in our patient was neither reported in literature nor in public databases. It affects the splice acceptor site of intron 9. Several bioinformatics tools (MaxEnt, NNSPLICE, HSF, SpliceSiteFinderLike, GeneSplicer, Hum Splicing Finder) predicted a strong effect on the splicing mechanism inducing exon 10 skipping. To investigate the effect of the splicing mutation on the transcript, we analyzed the SCN9A mRNA obtained from patient's whole blood. The potentially mis‐spliced region, from exon 9 to exon 11, was amplified by standard polymerase chain reaction. The fragments dimensional analysis on agarose gel showed the presence of two bands, one showing the expected length and a shorter one that was further characterized by Sanger sequencing. Differently from what predicted in silico, the exon 10 was not skipped but only partially deleted. Indeed, the nucleotide substitution in c.1108‐2 caused the displacement of the canonical acceptor splice site by 46 nucleotides within the exon 10. The loss of 46 nucleotides at the mRNA level (r.1108_1153del46) induced a shift in the reading frame, introducing a stop codon in position p.Gln369 (NP_002968).

The expression pattern of *SCN9A* in different cell types is still not fully defined. Interestingly, we have found sufficient traces of *SCN9A* transcript in whole blood sampling to allow a direct study of the mutation effect at the mRNA level, making redundant the in vitro model for the splicing mutation assay.

Skin biopsy analysis allowed expanding the features of the phenotype associated with this compound mutation. Indeed, we first demonstrated the lack of Meissner's mechanoreceptors, despite light touch sensation was normal as expected.^1^ Moreover, there was a severe denervation of the epidermis reflecting the loss of terminal nociceptors and explaining the lack of peripheral pain sensation. This finding is common to other inherited forms of CIP. Finally, structure and innervation density of cutaneous autonomic organs, including sweat glands, were normal differently from what expected in patients harboring mutations in NTRK1, NGFB, and PRDM12 genes in whom and anhydrosis is associated with CIP.^1^

Albeit overlapping, some phenotypic features might address the molecular diagnosis of CIP patients who, however, should be tested for all known genes when using next generation sequencing approach. *SCN9A* inactivating mutations should be searched in patients with anosmia, normal intelligence and normal^17^ or mildly abnormal^18^ large sensory nerve fiber function. In patients with normal intelligence showing a pattern of pain insensitivity restricted to the limbs rather than diffuse and no anosmia, mutations in *PRDM12* should be considered.^21^ CIP patients with normal intelligence, hyperhidrosis, gastrointestinal dysfunction and mild muscular weakness, without anosmia, neuropathy, or cardiovascular dysautonomia could harbor *SCN11A* mutations.^20^ When CIP is associated with impaired cognitive development and anhydrosis, the underlying molecular defect could be in the NTRK1 gene.^5^

We have identified and characterized a new molecular defect of *SCN9A* in a young patient with CIP, hyposmia and hypogeusia that widens the spectrum of Nav1.7 impairments underlying this phenotype, and have demonstrated the effect of the mutation on whole blood transcript assay.
